# Surrogacy Beyond Prognosis: The Importance of “Trial-Level” Surrogacy

**DOI:** 10.1093/oncolo/oyac006

**Published:** 2022-03-04

**Authors:** Marc Buyse, Everardo D Saad, Tomasz Burzykowski, Meredith M Regan, Christopher S Sweeney

**Affiliations:** 1 International Drug Development Institute, Louvain-la-Neuve, Belgium; 2 Interuniversity Institute for Biostatistics and statistical Bioinformatics (I-BioStat), Hasselt University, Hasselt, Belgium; 3 Dana Farber Cancer Institute, Boston, MA, USA

## Abstract

Many candidate surrogate endpoints are currently assessed using a 2-level statistical approach, which consists in checking whether (1) the potential surrogate is associated with the final endpoint in individual patients and (2) the effect of treatment on the surrogate can be used to reliably predict the effect of treatment on the final endpoint. In some situations, condition (1) is fulfilled but condition (2) is not. We use concepts of causal inference to explain this apparently paradoxical situation, illustrating this review with 2 contrasting examples in operable breast cancer: the example of pathological complete response (pCR) and that of disease-free survival (DFS). In a previous meta-analysis, pCR has been shown to be a strong and independent prognostic factor for event-free survival (EFS) and overall survival (OS) after neoadjuvant treatment of operable breast cancer. Yet, in randomized trials, the effects of experimental treatments on pCR have not translated into predictable effects on EFS or OS, making pCR an “individual-level” surrogate, but not a “trial-level” surrogate. In contrast, DFS has been shown to be an acceptable surrogate for OS at both the individual and trial levels in early, HER2-positive breast cancer. The distinction between the prognostic and predictive roles of a tentative surrogate, not always made in the literature, avoids unnecessary confusion and allows better understanding of what it takes to validate a surrogate endpoint that is truly able to replace a final endpoint.

## Teaser

Many candidate surrogate endpoints are currently assessed using a two-level statistical approach. This article explores tentative surrogates versus actual outcomes, focusing on the “surrogate paradox”.

**Implications for Practice**

The distinction between the prognostic and predictive roles of a tentative surrogate, not always made in the literature, avoids unnecessary confusion and allows better understanding of what it takes to validate a surrogate endpoint that is truly able to replace a final endpoint.

## Introduction

The search for surrogate endpoints in oncology has yielded interesting and informative results but also a large number of disappointments. Among the successes, disease-free (DFS) or relapse-free (RFS) survival were shown to be good surrogates for overall survival (OS) in the adjuvant treatment of colon cancer,^1^ gastric cancer,^2^ melanoma,^3^ and HER2-positive breast cancer,^4^ while metastasis-free survival was shown to be a good surrogate for OS in localized prostate cancer.^5^ Among the failures, pathological complete response (pCR) was not shown to be a good surrogate for event-free survival (EFS) after neoadjuvant treatment of operable breast cancer,^6^ while in advanced disease tumor response and progression-free survival (PFS) failed to be considered acceptable surrogates for OS in most^7-10^ (although not all^11,12^) solid tumors assessed thus far using meta-analyses of individual patient data (IPD).

All these studies used a so-called 2-level statistical approach to assess surrogacy, which relies on the availability of IPD.^13,14^ This approach consists in assessing whether 1) the potential surrogate is associated with the final endpoint (eg, OS) in individual patients and (2) the effect of treatment on the surrogate can be used to reliably predict the effect of treatment on the final endpoint. Both questions are of interest: the former for patient management (since a good surrogate is prognostic for the final endpoint) and the latter for drug development (since use of the surrogate instead of the final endpoint can lead to gains of months or even years of development time). Condition (1), called “individual-level surrogacy” or “patient-level surrogacy,” simply states that the surrogate is an independent prognostic factor for the final endpoint, and this can be tested in any series of patients, whether or not from a randomized trial. Condition (2), called “trial-level surrogacy” or “treatment-level surrogacy,” requires a meta-analysis of several randomized trials in which multiple estimates of treatment effects are available on both the surrogate and on the final endpoint.^15^ In all the examples cited, the condition of individual-level surrogacy was fulfilled, but some potential surrogates failed to meet the condition of trial-level surrogacy. Here we explore the following conceptual difficulty: how can the surrogate and the final endpoint be associated in individual patients, and yet treatment-induced improvements in the surrogate do not predict improvements in the final endpoint? Alternatively, this question can be phrased as “how is it possible that a tentative surrogate is prognostic for the final endpoint, a given treatment improves outcomes using the surrogate, and yet this same treatment does not significantly improve outcomes using the final endpoint?.” Of note, this conceptual difficulty is closely related to an extreme situation that has been called the “surrogate paradox” in the statistical literature, namely a situation in which treatment has a positive effect on the surrogate, the surrogate has a positive effect on the final endpoint, yet the treatment has a negative effect on the final endpoint.^16-18^ The example of pCR in operable breast cancer, which motivates this paper, is admittedly less extreme than in the surrogate paradox situation, but it has generated a lot of debate, if only because of its regulatory implications.^6,19^

## Causal Links Between the Surrogate and Final Endpoint

**Figure 1.**
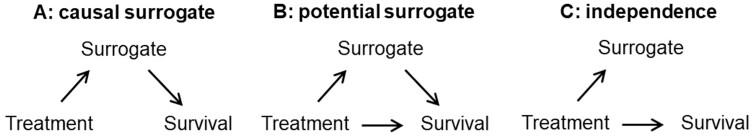
Causal diagrams illustrating pathways that involve treatment, a candidate surrogate, and the final end point, in this case survival (see text for explanations). Arrows indicate direct treatment effects: treatment effect on the surrogate, treatment effect on survival, and effect of surrogate on survival.

In order to serve as a surrogate, an intermediate endpoint should ideally be on the causal pathway between treatment and the final endpoint. The easiest way to understand this causal relation is to use diagrams inspired from causal inference (Fig. 1). For simplicity, we assume OS (simply, survival) to be the final endpoint in the remainder of this discussion.

Panel A in Fig. 1 shows an idealized situation of causal (or perfect) surrogacy, ie, all the treatment effect on survival is indirect, and mediated by the treatment effect on the surrogate (these effects are depicted as arrows). Panel B in Fig. 1 shows an intermediate situation where treatment has both a direct and an indirect effect on survival. Panel C in Fig. 1, in contrast, shows a situation where treatment has an effect on the surrogate and an effect on survival, but the 2 effects are completely independent of each other. Prentice built on the ideas captured in Fig. 1 to propose operational criteria for surrogate endpoint validation.^20^ In order to determine which of these 3 situations applies to a particular set of data, one would ideally want to estimate the proportion of treatment effect on survival that is mediated by the surrogate: this proportion would be equal to 100% for a causal (or perfect) surrogate (Panel A of Fig. 1), 0% for the situation of independence (Panel C of Fig. 1), or intermediate values otherwise (Panel B of Fig. 1). However, estimation of the proportion of treatment effect mediated—also known as the proportion explained^21^—has proven challenging statistically.^22^ The Prentice criteria remain conceptually important but have not led to convincing claims of surrogacy. The methods of causal inference can be used to estimate the proportion of treatment effect mediated, but their use requires untestable assumptions to be made, which may limit their utility in practice.^23^

## Explaining the Conceptual Difficulty

### Direct Treatment Effects

Let us return to our main question: how can a surrogate predict survival, and yet treatment-induced changes in the surrogate do not predict treatment-induced changes in survival? The first reason is apparent in Fig. 1, Panel B, where the treatment has a direct effect on survival, in addition to the indirect effect on survival that is mediated by the surrogate. In this situation, treatment effects on the surrogate would not fully translate to corresponding effects on survival. If direct treatment effects on survival were completely independent of the surrogate—say, a long-term risk of lethal toxicity—then improvements in the surrogate could still predict improvements in OS (through the indirect effect mediated by the surrogate). However, if the risk of lethal toxicity increased proportionately to the indirect effect mediated by the surrogate, then improvements in the surrogate might not lead to any benefit in OS.

### Confounding Factors

**Figure 2.**
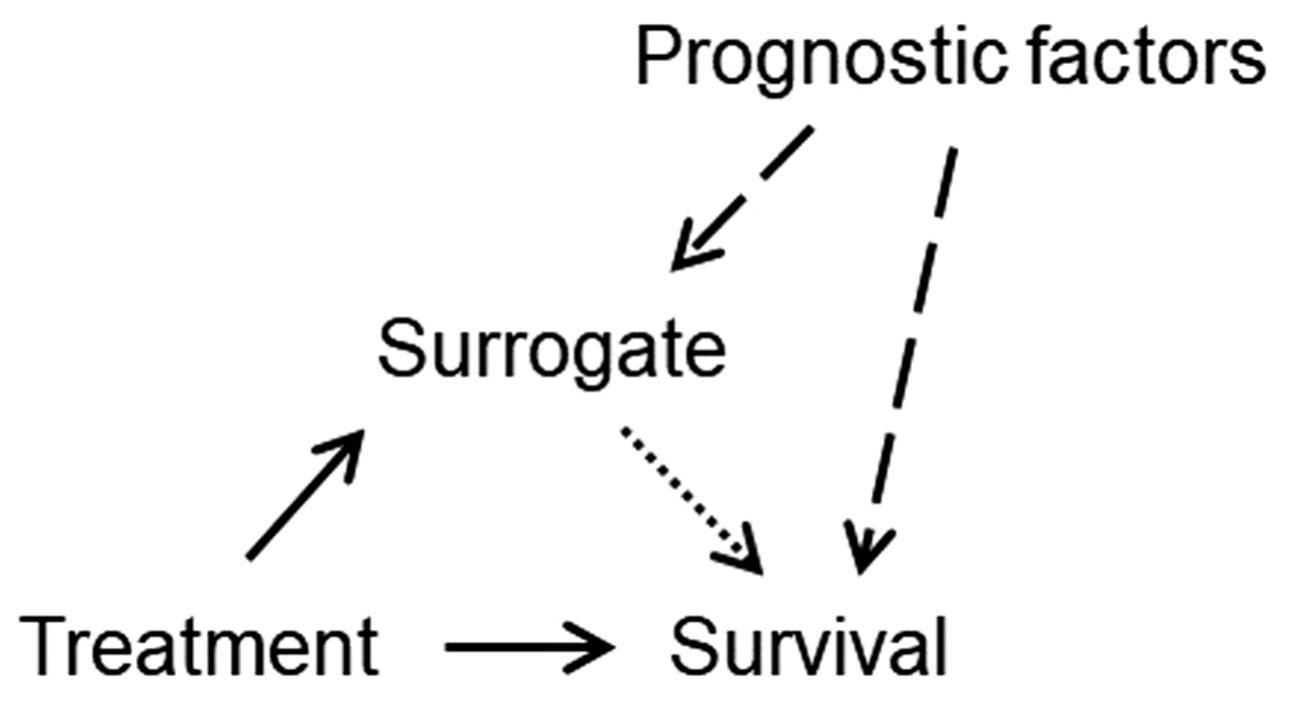
Prognostic factors for the surrogate and survival (dashed arrows) may create an apparent association between the surrogate and survival (dotted arrow), and hence an indirect effect of treatment on survival, even when direct treatment effects on the surrogate and survival are truly independent of each other.

A second reason for the conceptual difficulty discussed here is that the association between the surrogate and survival may be affected by confounding factors rather than to any causal mechanism. Figure 1 shows a simplified and unrealistic view of reality, since other factors than treatment often have an effect on the surrogate and on survival.^24^ However, this simplified view is sufficient to illustrate the key issue of confounding. Consider Fig. 2, identical to Fig. 1C (independence) but with the added impact of prognostic factors on the surrogate and on survival (dashed arrows).

Typically, the same factors tend to be prognostic for both the surrogate and survival, which creates an apparent correlation between the surrogate and survival at the individual level (dotted arrow): a low-risk patient will tend to have a good outcome on both the surrogate and survival, while a high-risk patient will tend to have a poor outcome on both the surrogate and survival. In the presence of such prognostic factors (known as confounders in causal inference), the correlation between the surrogate and survival will exist *even if* the treatment effects on the surrogate and survival are completely independent, as shown in Fig. 1C. In other words, the assessment of individual-level surrogacy is confounded by prognostic factors, known and unknown. One can adjust the analysis for known prognostic factors, but by definition one cannot adjust it for unknown prognostic factors. It is therefore a conceptual mistake to assume that if the surrogate and survival are correlated, a treatment-induced *change* in the surrogate (or lack thereof) will automatically induce a corresponding *change* in survival (or lack thereof), except under the implausible assumption of “no unknown confounders,” ie, all prognostic factors for the surrogate and for survival have been measured and accounted for in the analysis.

### Surrogate-Directed Treatment Changes

**Figure 3.**
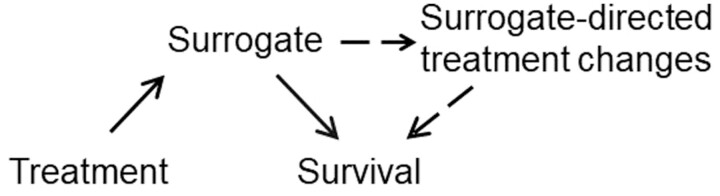
Surrogate-directed treatment changes may confound the trial-level association between the effects of initial treatment on the surrogate and on survival, even for a causal (perfect) surrogate.

The last and arguably most important reason for the conceptual difficulty is that observation of the surrogate itself will often induce a change in treatment which may, in turn, have an effect on survival. For example, physicians who treat patients with operable breast cancer may wish to use more aggressive adjuvant therapy after surgery for patients who fail to achieve a pCR after neoadjuvant therapy, than for those who do achieve a pCR. If more aggressive adjuvant therapy prolongs survival, then a treatment with a lower pCR rate may end up having the same effect on survival as a treatment with a higher pCR rate simply because of a higher proportion of patients receiving aggressive adjuvant therapy after surgery. Figure 3 illustrates how surrogate-directed treatment changes can confound the relationship between the surrogate and survival. Fig. 3 is identical to Fig. 1A (causal surrogate) but with the added impact of surrogate-directed treatment changes on survival (dashed arrows).

In the presence of such treatment changes, the trial-level association between the effects of the initial treatment on the surrogate and on survival may disappear *even if* the surrogate is causal (or perfect). The confounding effect of surrogate-directed treatment changes may be especially pronounced if potential surrogates are based on repeatedly measured biomarkers, such as minimal residual disease in hematological malignancies or circulating tumor DNA in solid tumors.

## Trial-Level Surrogacy

Given that individual-level surrogacy cannot predict how changes in the surrogate will translate into changes in survival in a group of patients, trial-level surrogacy is also required. Indeed, if multiple estimates of treatment effects on the surrogate are highly correlated with the corresponding estimates of treatment effects on survival, then it becomes possible to statistically predict—in a future trial—the effect of treatment on survival having observed the effect on the surrogate. Note that if such a high correlation has pragmatic value for prediction purposes, it does not guarantee that the surrogate is on the causal pathway of the treatment effect on survival. Note also that individual-level and trial-level surrogacy, being 2 distinct concepts, can be tested separately. Molenberghs and colleagues proposed a hierarchical model to test the 2 levels simultaneously.^15^ This model led to numerous developments since^13,14^ and has become an accepted way of assessing surrogacy when IPD from multiple randomized trials are available to do so.^25^ Nevertheless, the medical literature displays numerous instances in which trial-level surrogacy is essentially ignored in claims of surrogacy.^26-28^ A Cox model that estimates the impact of the surrogate on survival informs individual-level surrogacy; a meta-analysis of several randomized trials is required to inform trial-level surrogacy.

### Further Difficulties

Even in the best-case scenario where a meta-analysis of randomized trials addressing a specific therapeutic question can be conducted to test trial-level surrogacy, the results may not apply in a future trial testing a different question, for instance, the effects of a new drug with a novel mechanism of action, since the direct and indirect effects of such a drug on survival may be substantially different than with historical drugs. The increasing availability of active treatments after observation of the surrogate may also negatively impact trial-level surrogacy. For example, in patients with advanced colorectal cancer, PFS was a reasonable surrogate for survival when fluorouracil-based therapies were the only available second-line treatments: the trial-level coefficient of determination estimated from 10 randomized trials conducted in 1744 patients was *R*^2^ = 0.98 (95% confidence interval [CI]: 0.88 to 1.00^11^). In contrast, a more recent meta-analysis of 22 trials conducted in 16 762 patients found a much lower trial-level coefficient of determination *R*^2^ =0.46 (95% CI: 0.24 to 0.68^12^). Note that the confidence intervals around *R*^2^ can be wide, which implies that substantial uncertainty will typically affect predictions based on surrogates.

## Two Contrasting Examples in Breast Cancer

### Is pCR a Surrogate for EFS in Neoadjuvant Therapy?

It has been known for a long time that achieving pCR confers good prognosis in the neoadjuvant therapy of operable breast cancer.^29^ However, a treatment that improves the pCR rate does not necessarily improve long-term outcomes, such as DFS and OS, even in a trial in which patients achieving pCR had longer OS than patients not achieving pCR.^30,31^ Thus, the question remains: is this a trial specific or a general phenomenon? Using data from 12 randomized trials including 11 955 patients, Cortazar et al assessed pCR as a potential surrogate for EFS in patients undergoing neoadjuvant therapy for operable breast cancer (Table 1).^6^

**Table 1.** Two analyses assessing potential surrogates in breast cancer.

Setting	Surrogate	Final endpoint	No. of trials (no. of patients)	Known confounders	Trial-level *R*^2^ (95% CI)
Neoadjuvant therapy of operable disease^6^	pCR	EFS	12 trials (*N* = 11 955)	Tumor stage nodal status HR status HER2 status	0.03 (0.0-0.25)
Adjuvant anti-HER2 therapy^4^	DFS	OS	8 trials (*N* = 21 480)	Tumor stage nodal status HR status	0.85 (0.67-1.00)

CI, confidence interval; DFS, disease-free survival; EFS, event-free survival; HR, hormonal receptor; OS, overall survival; pCR, pathological complete response; *R*^2^, coefficient of determination.

Individual-level surrogacy was very strong across all trials: the hazard ratio for EFS of patients who reached pCR, when compared with those who did not, was 0.44 (95% confidence interval [CI], 0.39 to 0.51, unadjusted for baseline prognostic factors). In contrast, trial-level surrogacy was very weak, with a coefficient of determination R^2^=0.03 (95% CI: 0.0 to 0.25). This discrepancy between the strength of association at the individual-level and at the trial-level sparked enormous debate and controversy: there were only 12 trials in the meta-analysis, patients were not selected based on molecular subtypes, the treatment comparisons were very different from trial to trial, etc. Pointing to the limitations of Cortazar et al meta-analysis, some argued that pCR should be considered a surrogate based on the individual-level association only.^26^ As just discussed, this claim is unfounded regardless of the limitations of the meta-analysis. The US Food and Drug Administration has stated that pCR is a surrogate reasonably likely to predict long-term outcomes in these patients, and accepts pCR as an endpoint for accelerated approval; however, the agency still requires evidence of benefit on long-term outcomes to grant full approval.^19^ From the point of view of surrogacy, this position is ambiguous, as trial-level surrogacy has not been demonstrated^6^; from a clinical point of view, however, the position is tenable, and consistent with the need to tailor individual patient treatments according to whether they did or did not achieve a pCR (eg, to avoid post-operative adjuvant therapy in patients who achieved a pCR and to use aggressive post-operative adjuvant therapy in patients who did not). But, since there is no trial-level evidence of surrogacy for pCR, adjuvant treatment tailoring should be studied in randomized trials. The KATHERINE trial provides a perfect recent example of how individual responses can be used to modify adjuvant therapy in the hope of improving long-term outcomes.^32^

### Is DFS a Surrogate for OS in Adjuvant Therapy?

**Figure 4.**
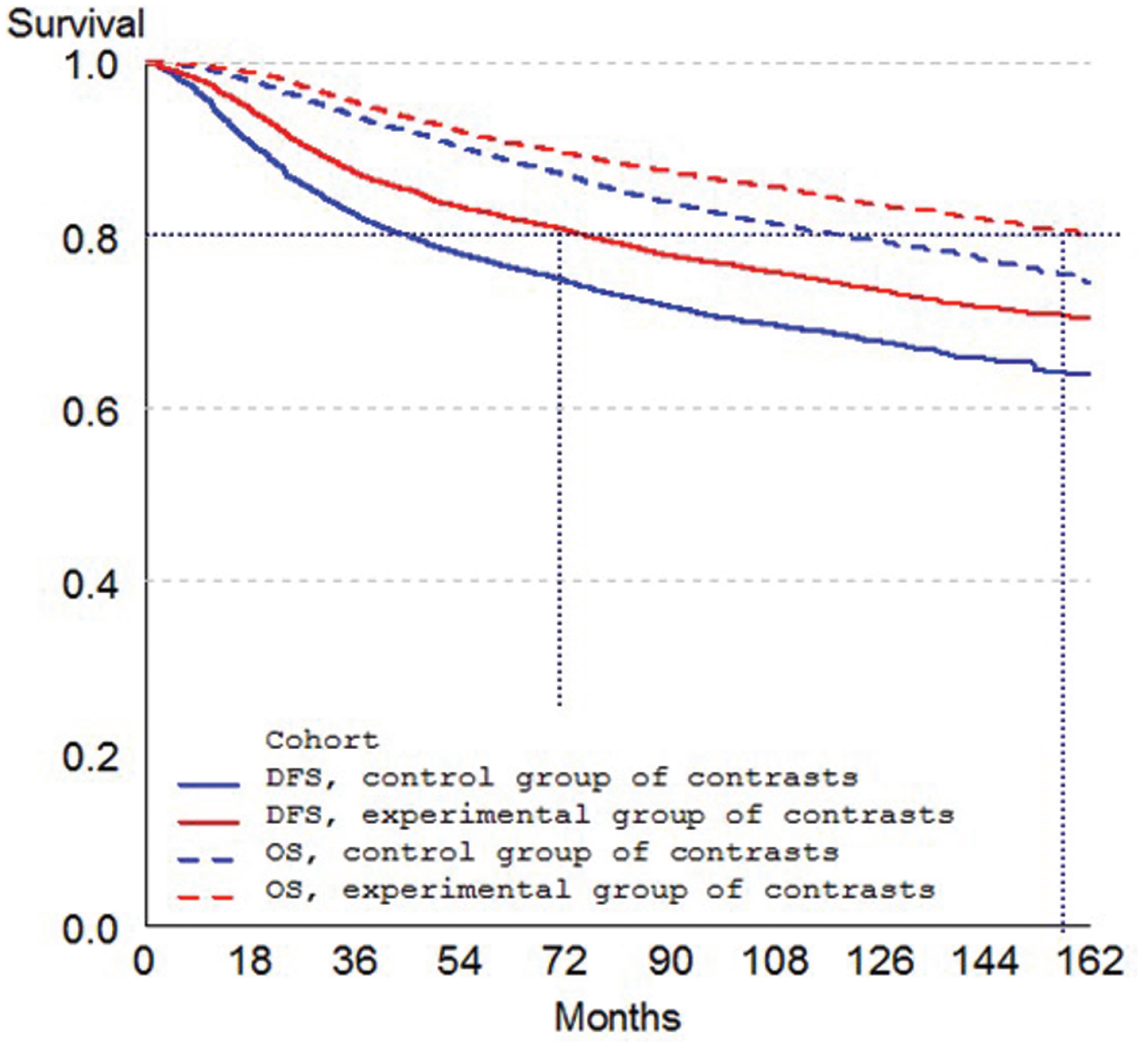
Kaplan-Meier curves of DFS (solid lines) and OS (dashed lines) in patients with HER2-positive operable breast cancer receiving adjuvant therapy with (red lines) or without (blue lines) trastuzumab.^4^

We assessed DFS as a surrogate for OS using data from 8 randomized trials including 21 480 patients in the setting of adjuvant (post-operative) anti-HER2 therapy for patients with early *HER2-*positive breast cancer (Table 1).^4^ Individual-level surrogacy was very strong: the correlation coefficient between DFS and OS was 0.90 (95% CI: 0.89 to 0.90, unadjusted for baseline prognostic factors). In this case, trial-level surrogacy was also very strong, with a coefficient of determination *R*^2^ = 0.85 (95% CI: 0.67 to 1.00) in an analysis that excluded one outlying trial and included 11 treatment contrasts. Based on these results, it is reasonable to continue to use DFS as a primary endpoint of clinical trials of adjuvant treatment for HER2-positive breast cancer. This result is especially useful given the long survival time of these patients. The Kaplan-Meier EFS and OS curves for this meta-analysis suggest that it takes approximately 72 months (6 years) to observe disease recurrence or death in 20% of patients, versus approximately 156 months (13 years) to observe death in 20% of patients; hence, using the surrogate might cut in half the time to the analysis of a randomized trial in this setting (Fig. 4). Such a gain in clinical development time justifies the search for surrogates, and the use of statistical methods to validate them. Of note, meta-analyses of patient-level data from all randomized trials are needed for this purpose, and data sharing will be essential for this purpose going forward.^33^

## Conclusion

Fleming and DeMets have famously stated that “a correlate does not a surrogate make.”^34^ This statement encapsulates the relatively frequent observation that candidate surrogates are usually acceptable at the individual (patient) level, but more rarely so at the trial (treatment) level.^6,8-10^ One may be tempted to try and find underlying biological explanations for this discrepancy in specific tumor types and treatment settings. Such biological explanations usually posit that some treatments may have a differential effect between primary tumors and metastases, thus offering a hypothesis to explain situations in which improvements in pCR, for example, are not associated with improvements in EFS or survival. However, the causal mechanisms summarized in Figs. 1 and 2 are sufficient to explain that the discrepancy can occur as a result of direct treatment effects, confounding by known and unknown prognostic factors, and surrogate-directed treatment changes. While the biological explanations may be plausible and even true in some cases, there is no statistical way to prove them with the data at hand, and so these explanations remain speculative as well as unnecessary to explain the observed data.

## Data Availability

The data underlying this article will be shared on reasonable request to the corresponding author.
